# Gut Microbiota Alterations and Dysbiosis Patterns in Pediatric Inflammatory Bowel Disease: Clinical Correlations and Therapeutic Impact

**DOI:** 10.3390/jcm15041589

**Published:** 2026-02-18

**Authors:** Anda-Maria Beca, Roxana Folescu, Adina Teodora Crăciun, Laura Olariu, Ileana Enatescu, Bianca Belei, Oana Belei

**Affiliations:** 1 Third Pediatric Clinic, “Louis Țurcanu” Emergency Children Hospital, 300041 Timișoara, Romania; anda.beca@rezident.umft.ro; 2 Department of Balneology, Medical Recovery and Rheumatology, Family Medicine Discipline, Center for Preventive Medicine, “Victor Babeș” University of Medicine and Pharmacy, 300041 Timisoara, Romania; 3 Department of Pediatrics, First Pediatric Clinic, “Victor Babeș” University of Medicine and Pharmacy, 300041 Timișoara, Romania; olariu.laura@umft.ro (L.O.); belei.oana@umft.ro (O.B.); 4 Twelfth Department, Neonatology Clinic, “Victor Babeș” University of Medicine and Pharmacy, 300041 Timișoara, Romania; enatescu.ileana@umft.ro; 5 Faculty of Medicine, “Victor Babeș” University of Medicine and Pharmacy, 300041 Timișoara, Romania; bianca.belei@student.umft.ro; 6 First Pediatric Clinic, Disturbances of Growth and Development on Children Research Center, “Victor Babeș” University of Medicine and Pharmacy, 300041 Timișoara, Romania

**Keywords:** pediatric inflammatory bowel disease, gut microbiota, dysbiosis, Crohn’s disease, ulcerative colitis, biologic therapy, intestinal microbiome, short-chain fatty acids, fecal microbiota analysis, microbial indices

## Abstract

**Background**: Gut microbiota alterations are increasingly recognized as key contributors to the development and clinical course of inflammatory bowel disease (IBD), particularly in pediatric patients, in whom microbial maturation and immune regulation are still evolving. **Objective**: This study aimed to assess intestinal microbiota composition and dysbiosis severity in pediatric IBD, with comparative analyses according to disease phenotype (Crohn’s disease versus ulcerative colitis) and therapeutic strategy (biologic versus non-biologic treatment). **Methods**: A prospective cohort of 60 pediatric patients diagnosed with IBD based on Porto criteria was evaluated. Fecal samples were obtained at baseline and after three months of combined standard IBD treatment and adjunct microbiota-targeted therapy, and were analyzed using an AI-assisted microbiota profiling platform. A semi-quantitative dysbiosis score was calculated based on the relative abundance of proinflammatory taxa and depletion of short-chain fatty acid (SCFA)-producing bacteria. Microbial parameters were correlated with clinical and therapeutic variables, including the Organism of Interest metric and the Gut Microbiota Index (GMI). **Results**: Dysbiosis severity was significantly higher in patients with Crohn’s disease compared with ulcerative colitis (9.65 ± 1.44 vs. 8.42 ± 1.88, *p* = 0.037). Patients receiving biologic therapy showed a trend toward lower dysbiosis scores and improved microbial indices, although statistical significance was not reached. Severe dysbiosis was identified in 46.7% of the cohort. Strong positive correlations were observed between the dysbiosis score, Organism of Interest metric and GMI (*r* = 0.68–0.72, *p* < 0.01). **Conclusions**: Pediatric IBD is associated with a reproducible dysbiotic profile, more pronounced in Crohn’s disease and partially modulated by biologic therapy. The observed correlations between microbiota-derived indices support their potential utility as complementary markers of intestinal microbial imbalance and disease activity.

## 1. Introduction

The development of the gut microbiota during early life has a profound influence on health outcomes later in life. Early environmental exposures, beginning in utero and extending through infancy, shape the maturation and function of the intestinal microbiota, with long-lasting effects on immune regulation, metabolic balance and overall host physiology [1]. A substantial proportion of the gut microbial community, estimated at 30 to 40%, remains uncultured or unidentified, highlighting the complexity of this ecosystem [1]. Trillions of microorganisms colonize the mucosal surfaces of the human body and contribute to essential physiological and pathophysiological processes throughout life [2].

A balanced intestinal microbiota supports mucosal healing and maintains epithelial barrier integrity, whereas dysbiosis, defined as the disruption of normal microbial composition, has been implicated in a wide range of inflammatory and metabolic disorders [3]. In healthy individuals, the gut microbiota is dominated by members of the Firmicutes and Bacteroidetes phyla [4], and more than 2000 bacterial species have been described, with most belonging to *Firmicutes*, *Bacteroidetes*, *Actinobacteria* and *Proteobacteria* [5]. Despite this diversity, interpersonal variability is high, and microbial communities often cluster into distinct enterotypes dominated by *Bacteroides*, *Prevotella* or *Ruminococcus* [6].

In inflammatory bowel disease (IBD), most studies report an increased abundance of *Actinobacteria* and *Proteobacteria* together with a reduction in *Firmicutes* and *Bacteroidetes*, reflecting a shift toward a proinflammatory microbial profile [3,7]. Microbial diversity is typically reduced in IBD, particularly among beneficial short-chain fatty acid (SCFA)-producing families such as *Ruminococcaceae* and *Lachnospiraceae*, whereas proinflammatory taxa including *Enterobacteriaceae* and *Fusobacteriaceae* are expanded [8,9]. This imbalance between anti-inflammatory and proinflammatory bacteria contributes directly to intestinal inflammation [10], partly by altering epithelial barrier integrity. Increased permeability and disrupted tight junction protein expression, characteristic of the “leaky gut” phenotype, facilitate the translocation of microbial products and sustain inflammatory cascades [11].

Over recent decades, IBD has become a global health concern, with rapidly rising incidence in newly industrialized and Westernizing societies [12]. Among pediatric patients, 4% present before the age of five years and 18% before the age of ten, with peak onset during adolescence [13].

In pediatric populations, Crohn’s disease and ulcerative colitis differ with respect to disease distribution, clinical behavior, and therapeutic management. Crohn’s disease is characterized by transmural inflammation and may affect any segment of the gastrointestinal tract, whereas ulcerative colitis is confined to the colon and rectum and is associated with continuous mucosal inflammation. Therapeutic approaches in pediatric IBD include non-biologic treatments such as aminosalicylates, corticosteroids, and immunomodulators, as well as biologic agents, primarily anti–tumor necrosis factor therapies, which are increasingly employed in moderate to severe disease to induce and maintain remission.

Although its precise pathogenesis remains incompletely understood, IBD is believed to arise from the interplay of environmental risk factors, genetic susceptibility and aberrant immune responses directed against the host’s own intestinal microbiota [14]. In pediatric populations, disease mechanisms involve complex interactions between microbial dysbiosis, environmental triggers, host genetic factors and immune dysregulation [15,16]. Genetic susceptibility can influence the structure and stability of the intestinal microbiota, further predisposing individuals to dysregulated immune responses [17].

Dysbiosis in pediatric IBD is characterized by reductions in beneficial SCFA-producing bacteria, including *Faecalibacterium prausnitzii*, *Blautia*, *Roseburia* and several members of the *Ruminococcaceae* and *Lachnospiraceae* families, together with increases in *Enterococcus*, *Proteobacteria* and other pathobionts [16,18]. Similar microbial alterations have been described in pediatric Crohn’s disease cohorts, with enrichment of Proteobacteria and depletion of Clostridiales and key butyrate-producing taxa such as F. prausnitzii [19]. SCFAs, particularly butyrate, play a central role in maintaining epithelial homeostasis by serving as a primary energy source for colonocytes and by exerting anti-inflammatory effects [20]. Reduced SCFA production and decreased butyrate levels have been consistently documented in IBD patients [18,21], and impaired butyrate metabolism has been proposed as a mechanistic contributor to ulcerative colitis pathogenesis [22]. *F. prausnitzii*, one of the most abundant butyrate-producing species in the healthy gut microbiota, is markedly depleted in IBD and has attracted considerable interest due to its anti-inflammatory properties and potential therapeutic relevance [23].

Although the causal role of the microbiome in IBD remains unresolved, the strong association between microbial imbalance, epithelial barrier dysfunction and inflammatory activation underscores the importance of microbiota-focused research [5,16,20]. Targeting dysbiosis as a personalized therapeutic strategy has gained increasing attention, although further well-designed prospective studies are required to clarify optimal approaches [24]. Microbial signatures also hold promise as predictive biomarkers of treatment response and disease monitoring, representing an emerging area of investigation in pediatric IBD [25].

Pediatric IBD represents a distinct clinical entity, in which early-life modulation of the gut microbiota may have long-term implications for disease course, maintenance of remission, and transition into adulthood. Given these complex interactions, characterizing microbiota structure in pediatric IBD may provide valuable insights into disease mechanisms, therapeutic effects and potential biomarkers of disease activity. The present study aimed to evaluate intestinal dysbiosis and microbial imbalance in pediatric Crohn’s disease and ulcerative colitis using quantitative microbial indices and organism specific profiling. Furthermore, it assessed the influence of biologic therapy and sodium butyrate supplementation on microbial composition, with the objective of identifying microbiota related indicators of disease activity and treatment response.

## 2. Materials and Methods

This prospective observational study was conducted over a three month period and included 60 pediatric patients diagnosed with inflammatory bowel disease (IBD) according to the Porto criteria for the classification and evaluation of pediatric IBD. Participants were at different stages of disease evolution and were undergoing various therapeutic regimens, including newly diagnosed patients and those receiving immunosuppressive or biologic therapy.

The study population was analyzed according to predefined clinical and therapeutic subgroups. Patients were stratified by diagnosis into Crohn’s disease (*n* = 36) and ulcerative colitis (*n* = 24). Additional subgroup analyses were performed based on treatment exposure, including biologic therapy versus non biologic therapy, as well as adjunct microbiota targeted interventions. These subgroup comparisons were predefined and used consistently throughout the statistical analysis.

Study design and sample collection. Each patient provided two fecal samples: one collected at baseline (before any microbiota-targeted intervention) and a second sample collected after three months of therapy. All samples were processed in a reference laboratory using an artificial intelligence based microbial profiling platform capable of identifying clinically relevant bacterial species associated with intestinal inflammation and dysbiosis. After collection, stool samples were processed according to standardized laboratory protocols at the reference facility, with microbial DNA extraction and 16S rRNA sequencing followed by AI-assisted classification and pattern recognition using the NostraBiome proprietary platform (NostraBiome, Arad, Romania), according to the analytical workflow of the reference laboratory, to identify dysbiosis patterns and quantify clinically relevant bacterial taxa. A detailed description of the AI-assisted microbiota profiling workflow is provided in the Supplementary Materials (Supplementary Material S1).

Both baseline and post treatment microbiota results were available for all patients. The first sample reflected the pretreatment microbial status, while the second sample allowed direct comparison of microbiota composition after therapy. The analysis generated two quantitative microbial indices, the Gut Microbiota Index (GMI) and the Organism of Interest metric, which were evaluated before and after treatment to assess microbial changes over time.

This approach enabled the characterization of baseline dysbiosis and the evaluation of microbial response following standard therapeutic interventions and microbiota targeted therapy.

Therapeutic interventions. Therapeutic management was not modified for study purposes and followed standard clinical practice. In addition to standard IBD therapy, patients received adjunct microbiota targeted interventions based on individual microbiota profiles, including sodium butyrate supplementation and, when clinically indicated, probiotics, prebiotics, or antibiotics. This design allowed the assessment of the global microbial response to integrated clinical management.

Clinical and laboratory data. Clinical data included age, sex, mode of delivery, early feeding pattern, diagnosis (Crohn’s disease or ulcerative colitis), and ongoing treatment. Microbial data were extracted from laboratory reports issued by the external reference facility.

The following pathobiont species were identified and included in the analysis: *Bacteroides fragilis*, *Bilophila wadsworthia*, *Clostridioides difficile*, *Ruminococcus gnavus*, *Ruminococcus torques*, *Eggerthella lenta*, *Desulfovibrio* spp., *Klebsiella* spp., *Escherichia coli*, *Campylobacter* spp., *Fusobacterium* spp., and *Clostridium perfringens*.

Inflammatory markers, including C-reactive protein, erythrocyte sedimentation rate, and fecal calprotectin, were recorded as part of routine clinical assessment. These variables did not show significant correlations with the microbiota-derived indices in this cohort and were therefore not included as primary outcomes in the analysis.

Dysbiosis score calculation. A semi quantitative dysbiosis score was calculated for each patient based on the presence of proinflammatory species and the absence of beneficial short chain fatty acid-producing taxa. Each proinflammatory species contributed 1 point, and the absence of each protective species contributed an additional point. Higher scores indicated more severe microbial imbalance. This score was used to compare patient subgroups (Crohn’s disease vs. ulcerative colitis, biologic vs. non-biologic therapy) and to explore associations between microbial imbalance and clinical markers.

Statistical analysis. Descriptive and inferential statistics were performed according to data distribution. Continuous variables are presented as mean ± standard deviation for descriptive and comparative purposes, although non-parametric tests were applied due to deviations from normal distribution. Normality of continuous variables (age, dysbiosis score, number of pathobionts) was evaluated using the Shapiro–Wilk test. As most variables were non normally distributed, comparisons between independent groups (Crohn’s disease vs. ulcerative colitis; biologic vs. non biologic therapy) were performed using the Mann–Whitney U test. Categorical variables (sex, diagnosis, treatment type, presence of bacterial species) were analyzed using the chi-square test or Fisher’s exact test when appropriate. Correlations between dysbiosis scores and inflammatory markers were assessed using the Spearman correlation coefficient. A *p* value below 0.05 was considered statistically significant.

Statistical analyses were performed using IBM SPSS Statistics for Windows, Version 26.0 (IBM Corp., Armonk, NY, USA).

## 3. Results

### 3.1. Clinical Results and Dysbiosis Score

The study included 60 pediatric patients diagnosed with inflammatory bowel disease (IBD), with a mean age of 13.7 years. Of these, 38 were male (63.3%) and 22 were female (36.7%). Regarding the mode of delivery, 32 children (53.3%) were delivered by cesarean section, while 28 (46.7%) were born vaginally.

Crohn’s disease (CD) was more frequent, being diagnosed in 36 patients (60%), whereas ulcerative colitis (UC) was identified in 24 patients (40%). Early-life feeding patterns varied: 28 children (46.7%) received mixed feeding (breast milk and formula), 15 (25%) were exclusively breastfed, 15 (25%) were exclusively formula-fed, and 2 children (3.3%) received breast milk combined with cow’s milk.

Regarding treatment, 17 patients (28.3%) were receiving biologic therapy, 17 (28.3%) azathioprine, and 16 (26.7%) a combination of both. Smaller groups included patients treated with 5-ASA (*n* = 3), corticosteroids (*n* = 3), or a combination of 5-ASA and corticosteroids (*n* = 3). One patient was receiving triple therapy consisting of a biologic agent, azathioprine, and 5-ASA.

Table 1 summarizes the demographic and clinical characteristics of the cohort.

Analysis of the dysbiosis score revealed clear differences between diagnostic and therapeutic subgroups.

Patients with Crohn’s disease showed higher mean dysbiosis scores (9.65) compared with those with ulcerative colitis (8.42), indicating more pronounced microbial imbalance in CD.

When evaluating treatment effects, patients receiving biologic therapy had a lower mean dysbiosis score (8.8) compared with those not treated with biologics (9.5), suggesting a potential beneficial impact of biologics on microbiota composition.

Within the Crohn’s subgroup, patients without biologic therapy demonstrated higher dysbiosis scores (9.78) compared with those receiving biologics (9.56). In contrast, among UC patients, the mean dysbiosis score was slightly higher in those treated with biologics (9.0) than in those without biologics (8.0), although this difference was not statistically significant. These findings suggest that the microbiota-modulating effect of biologic therapy may be more pronounced in Crohn’s disease.

Corticosteroid therapy was not associated with meaningful differences in dysbiosis score (9.3 in patients receiving corticosteroids versus 9.0 in the remaining cohort).

Feeding patterns were also linked to microbial composition: exclusively breastfed children exhibited lower dysbiosis scores (~8.6) than those fed formula (~9.4) or mixed feeding (~9.1), supporting the protective role of breastfeeding in microbiota development.

A detailed overview of dysbiosis score comparisons across clinical categories is provided in Table 2.

### 3.2. Microbiological Analysis and Bacterial Species Distribution

The intestinal microbiota analysis included 60 pediatric patients, of whom 36 were diagnosed with Crohn’s disease (CD) and 24 with ulcerative colitis (UC). The proportion of patients in whom at least one potentially pathogenic bacterial species was identified was high in both entities—94% in CD and 92% in UC.

The mean number of pathobionts per patient was slightly higher in Crohn’s disease compared with ulcerative colitis (2.76 ± 1.44 vs. 2.50 ± 1.88), suggesting a more pronounced degree of dysbiosis in CD. When treatment exposure was considered, patients without biologic therapy had, on average, more pathobionts per patient (2.87 ± 1.55) than those receiving biologic agents (2.43 ± 1.70). This trend persisted after stratification by diagnosis. In Crohn’s disease, non-biologic patients had a mean of 3.00 pathobionts per patient, compared with 2.56 among those treated with biologics. In ulcerative colitis, non-biologic patients had a mean of 2.71 pathobionts per patient, compared with 2.20 in biologic-treated patients. A summary of the mean number of pathobionts across diagnostic and therapeutic subgroups is presented in Table 3.

At the species level, *Bacteroides fragilis* was the most frequently identified organism (detected in 34 patients), followed by *Bilophila wadsworthia* and *Clostridioides difficile* (each found in 20 patients). *Eggerthella lenta* was identified in 12 patients, and *Ruminococcus torques* in 8 patients.

**Figure 1:**
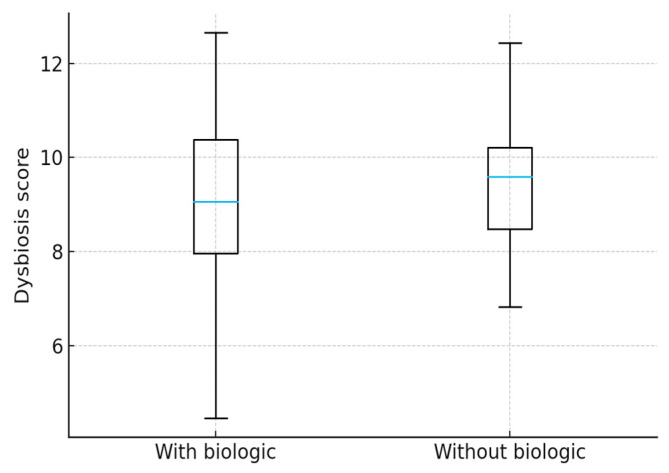
Distribution of dysbiosis scores in pediatric IBD patients receiving biologic versus non-biologic therapy.

The bacterial profile differed between the two IBD subtypes. Patients with Crohn’s disease more frequently harbored *Bilophila wadsworthia* and *Clostridioides difficile*, whereas those with ulcerative colitis showed a predominance of *Ruminococcus torques* and *Eggerthella lenta*. These findings indicate distinct microbial signatures between Crohn’s disease and ulcerative colitis. Overall, the results support an association between Crohn’s disease and a more severe dysbiotic pattern, characterized by a higher burden of pro-inflammatory bacterial species. Biologic therapy appears to contribute to a reduction in microbial imbalance, although this effect was modest, possibly influenced by baseline disease severity and the limited size of the treatment subgroups. The distribution of pathobiont burden according to treatment exposure is illustrated in Figure 1.

### 3.3. Associations Between Diagnosis, Treatment, and Bacterial Profile

A chi-square (χ^2^) test was performed to assess associations between disease type (Crohn’s disease vs. ulcerative colitis), biologic therapy, and the presence of the main bacterial species identified. A detailed summary of these statistical results is provided in Table 4.

The distribution of bacterial taxa differed significantly between the two IBD subtypes. *Bilophila wadsworthia* (*p* = 0.024) and *Clostridioides difficile* (*p* = 0.032) were detected significantly more often in patients with Crohn’s disease, whereas *Ruminococcus torques* was more frequently identified in ulcerative colitis (*p* = 0.044). Regarding biologic therapy, no statistically significant differences were observed between treated and untreated patients (*p* > 0.05). The bacterial distributions were comparable, suggesting that the impact of biologic therapy on microbial composition was modest.

Analysis of the relationship between an increased number of pathobionts (≥ 3) and disease type revealed a significant association (*p* = 0.028), with a higher frequency of severe dysbiosis in Crohn’s disease compared with ulcerative colitis. Conversely, the relationship between a higher number of pathobionts and biologic treatment was not statistically significant (*p* = 0.35), although a trend toward lower dysbiosis prevalence was observed among biologic-treated patients.

Overall, the chi-square analysis confirmed the association between Crohn’s disease and the presence of pro-inflammatory bacteria, particularly *Bilophila wadsworthia* and *Clostridioides difficile*, whereas *Ruminococcus torques* was more frequently associated with ulcerative colitis. Although biologic therapy did not significantly alter the distribution of pathobionts, the general trend suggested a moderate protective effect on intestinal microbial balance.

### 3.4. Microbial Index Analysis

To further characterize intestinal dysbiosis, two quantitative microbiome-derived indices, the Organism of Interest score and the Gut Microbiome Index *(GMI)*, were assessed across the full cohort of 60 pediatric IBD patients. Their distribution patterns closely mirrored those observed for the dysbiosis score, reinforcing the consistency of microbiota-based alterations within the study population.

Between diagnostic subgroups, both indices showed comparable mean values, with no statistically significant differences (*p* > 0.05). Nevertheless, patients with Crohn’s disease exhibited slightly higher average scores than those with ulcerative colitis, suggesting a tendency toward more pronounced microbial imbalance. These comparative values are summarized in Table 5.

In contrast, biologic therapy was associated with significantly lower microbial index values compared with non-biologic therapy (*p* < 0.05 for both indices), indicating a potential protective or stabilizing effect of biologic agents on intestinal microbial homeostasis. Detailed results are presented in Table 6.

A strong positive correlation was found between the Organism of Interest score and the GMI (Spearman’s r = 0.68, *p* < 0.01), confirming that these indices measure related aspects of dysbiosis severity and reinforce each other’s validity as microbiome health markers. Correlation metrics are shown in Table 7.

These results underscore the robustness of the microbial indices and support their potential integration into additional analyses, especially those assessing dysbiosis severity and clinical progression.

### 3.5. Summary of Dysbiosis and Microbial Correlations

The intestinal dysbiosis score and quantitative microbial indices were evaluated in all 60 pediatric patients with inflammatory bowel disease (IBD).

**Figure 2:**
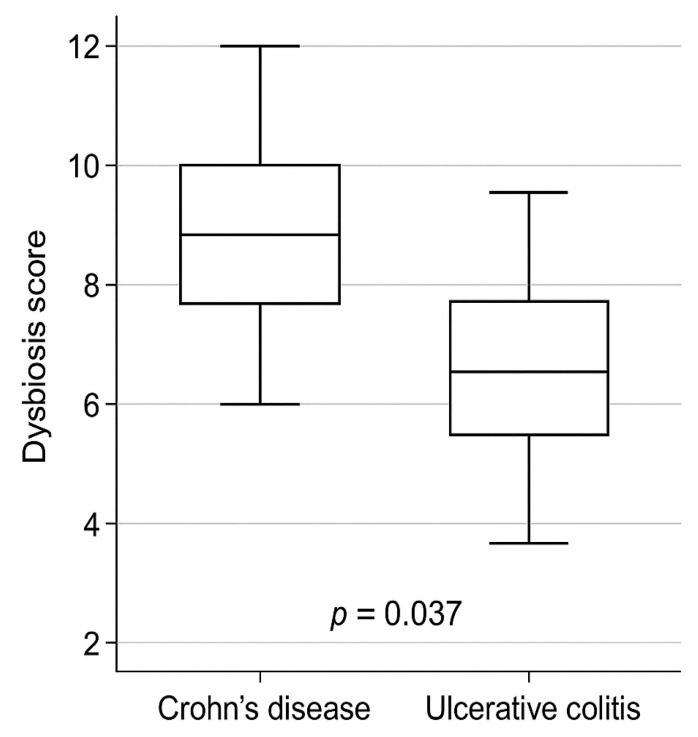
Dysbiosis score by diagnosis.

Patients with Crohn’s disease (CD) exhibited significantly higher dysbiosis scores compared with those with ulcerative colitis (UC) (9.65 ± 1.44 vs 8.42 ± 1.88, *p* = 0.037), indicating a more pronounced microbial imbalance. These results are summarized in Table 8 and illustrated in Figure 2.

Although the difference between patients receiving biologic therapy and those without biologic treatment did not reach statistical significance (*p* = 0.17), a tendency toward lower dysbiosis scores was observed among biologic-treated patients. The data are provided in Table 9.

Regarding dysbiosis severity, nearly half of the cohort (46.7%) presented with severe imbalance, most frequently observed in Crohn’s disease. Distribution values are shown in Table 10.

The microbial indices, Organism of Interest and GMI, showed comparable values between CD and UC, with slightly higher levels in CD. These comparisons are presented in Table 11.

Both microbial indices showed strong correlations with the dysbiosis score, confirming their consistency and shared biological meaning. Correlation values are shown in Table 12.

In short, microbial indices demonstrated strong coherence with the dysbiosis score, reinforcing their reliability as markers of microbial imbalance. Together, these results support the integration of quantitative microbiome-derived indices into clinical and research assessments of dysbiosis severity and IBD progression.

### 3.6. Effect of Butyrate Therapy on Microbial Indices

**Figure 3:**
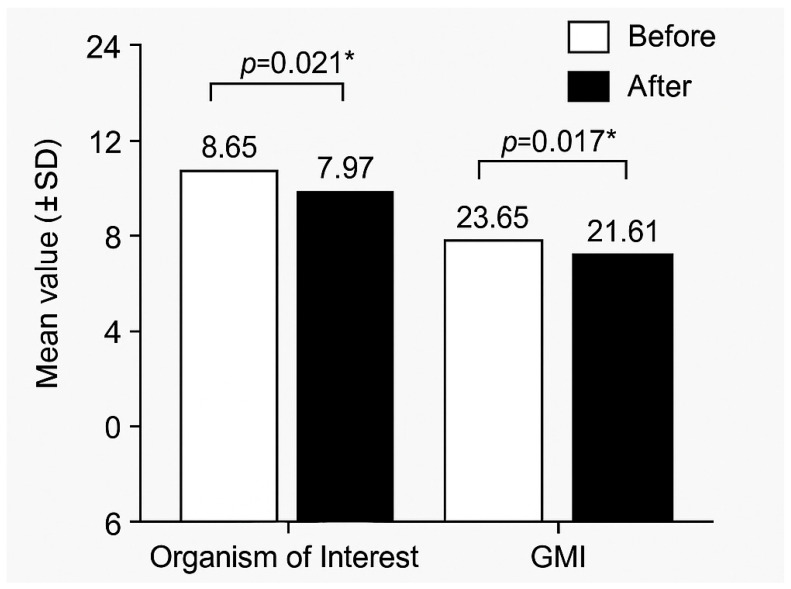
Evolution of microbial indices before and after butyrate therapy. * *p* < 0.05 was considered statistically significant.

After three months of sodium butyrate therapy, both microbial indices (Organism of Interest and GMI) decreased significantly, indicating partial restoration of intestinal microbial balance. The quantitative evolution is summarized in Table 13 and visualized in Figure 3.

The improvement was more pronounced in Crohn’s disease compared with ulcerative colitis. These subgroup differences are shown in Table 14.

Patients receiving biologic therapy exhibited greater improvement in microbial indices compared with those not treated with biologics, suggesting a potential synergistic effect between biologic agents and butyrate supplementation. The results are shown in Table 15.

### 3.7. Interpretative Summary

Intestinal dysbiosis was more pronounced in Crohn’s disease, reinforcing disease-specific patterns of microbial disruption. Biologic therapy tended to be associated with lower dysbiosis and lower microbial index values, indicating a modest but favorable modulatory effect on gut microbial composition. Nearly half of the cohort presented with severe dysbiosis, emphasizing the extent of microbial imbalance in pediatric IBD.

Both microbial indices (Organism of Interest and GMI) showed strong correlations with the dysbiosis score, supporting their reliability as consistent measures of intestinal microbial health. Their concordance highlights their potential clinical utility as complementary tools for the assessment of microbial imbalance.

Overall, these findings demonstrate a reproducible pattern of dysbiosis in pediatric IBD, more marked in Crohn’s disease and partially mitigated by biologic therapy. Improvements observed after butyrate supplementation further support the use of microbiota-targeted interventions. These results form a coherent basis for the subsequent discussion, which explores their biological relevance, consistency with the current literature, and implications for clinical management.

## 4. Discussion

The present study provides further insight into the patterns of intestinal dysbiosis characterizing pediatric inflammatory bowel disease and their modulation under different therapeutic strategies. Our findings indicate that disease phenotype significantly influences dysbiosis severity, with Crohn’s disease showing a more profound disruption of microbial homeostasis compared with ulcerative colitis. This finding supports the concept that disease phenotype influences the extent of microbiota alteration and reinforces the relevance of dysbiosis severity as a disease-specific feature in pediatric IBD. Consistent with previous findings, we observed a pronounced dysbiosis across the cohort, with significantly higher dysbiosis scores in Crohn’s disease than in ulcerative colitis, a pattern widely reported in pediatric IBD populations [16,18,19]. This imbalance, reflected by reduced short chain fatty acid (SCFA)-producing taxa such as *Faecalibacterium prausnitzii*, *Blautia*, *Roseburia* and several members of the Ruminococcaceae and Lachnospiraceae families, together with increased abundances of *Enterococcus* and Proteobacteria, aligns with established evidence linking dysbiosis to impaired epithelial barrier function and enhanced mucosal inflammation [11,16].

The significant correlations identified between the dysbiosis score, the Organism of Interest metric and the GMI support their value as complementary quantitative markers of microbial imbalance. Similar relationships have been reported in previous studies, where decreased microbial diversity and depleted Firmicutes were associated with heightened inflammatory burden and more active disease [9,20,26]. Pediatric data also indicate that steroid non-responders display less diverse microbiota than responders, highlighting the prognostic relevance of baseline microbial characteristics [27].

The concordance observed among these microbiota-derived indices suggests that they capture complementary dimensions of dysbiosis severity and may provide a robust and clinically meaningful assessment of microbial imbalance. From a clinical perspective, their consistent behavior supports potential utility as adjunctive tools for monitoring microbial status alongside conventional disease activity measures.

Although biologic therapy was associated with lower dysbiosis scores in our cohort, these differences did not reach statistical significance. Previous work has shown that anti-TNF agents may partially restore microbial diversity. Carlsen et al. demonstrated that gut diversity progressively declined as the interval since the last infliximab infusion increased, mirroring rising disease activity and diminishing SCFA-producing taxa [20].

In our cohort, the microbiota-modulating effect of biologic therapy appeared more evident in Crohn’s disease than in ulcerative colitis, which may reflect differences in disease behavior, inflammatory burden, and baseline dysbiosis severity. These findings suggest that biologic therapy may exert indirect effects on microbial composition primarily through inflammation control, rather than acting as a direct microbiota-targeted intervention. Other pediatric studies have reported increases in biodiversity and shifts toward healthier microbial profiles following infliximab initiation [28]. Specific microbial signatures associated with favorable treatment response have also been described, including higher abundances of *Bifidobacterium*, *Collinsella*, *Lachnospira*, various Lachnospiraceae, *Roseburia* and *Eggerthella*, along with reduced *Phascolarctobacterium* in anti-TNF responders [26]. *Faecalibacterium prausnitzii* has additionally been proposed as a predictor of recurrence after infliximab discontinuation [26]. Patients receiving biologic therapy have also been shown to exhibit lower baseline Firmicutes and *Mycoplasma hominis* levels relative to conventionally treated individuals [29].

Recent microbiome and metabolomics-focused studies suggest that assessing microbial structure and metabolic function may provide valuable insights for optimizing anti-TNF therapy, particularly by identifying microbial signatures associated with treatment responsiveness [30].

Our findings are also consistent with earlier reports documenting expansion of Proteobacteria and Enterobacteriaceae, along with marked depletion of *F. prausnitzii*, a key butyrate-producing species with immunomodulatory and epithelial protective properties [21,23]. Impaired butyrate metabolism represents a fundamental pathogenic mechanism in both Crohn’s disease and ulcerative colitis [18,22]. Consistent reductions in fecal butyrate levels have been widely reported among IBD patients [21], while loss of SCFA-producing Firmicutes, such as *Faecalibacterium*, *Ruminococcus*, *Roseburia* and *Clostridium* clusters IV and XIVb, has been linked to increased inflammatory activity [26].

In our cohort, sodium butyrate supplementation was associated with a significant decrease in microbiota-derived indices over the three-month follow-up period, suggesting partial restoration of intestinal microbial balance. The observed improvement was more pronounced in patients with Crohn’s disease and in those receiving concomitant biologic therapy, indicating a potential synergistic effect between anti-inflammatory treatment and microbiota-targeted modulation. These findings support the biological relevance of short-chain fatty acid pathways in maintaining intestinal homeostasis in pediatric IBD. Beyond global diversity loss, specific microbial alterations have been strongly associated with disease phenotype. Newly diagnosed pediatric Crohn’s disease patients frequently exhibit increased Enterobacteriaceae, *Fusobacteriaceae*, *Pasteurellaceae* and *Veillonellaceae*, together with reductions in *Bacteroidales*, *Clostridiales* and *Erysipelotrichales* [19,26]. Active inflammation induces substantial shifts in microbial composition, some of which may partially revert during remission, especially in response to anti-TNF therapy [31]. Decreased abundances of *Ruminococcaceae* and *Lachnospiraceae*, both essential butyrate producers, are considered hallmarks of dysbiosis associated with Crohn’s disease [32]. Increased Actinobacteria and Proteobacteria, along with reduced Firmicutes, have also been correlated with greater IBD activity [33].

Several taxa implicated in disease mechanisms have been identified. *Fusobacterium varium*, commonly detected in ulcerative colitis mucosa, has been proposed as a potential pathogenic contributor [34]. Although *Bacteroides fragilis* is generally regarded as beneficial, certain strains can invade epithelial tissues and contribute to mucosal injury in some ulcerative colitis patients [34]. Transient blooms of *Ruminococcus gnavus* are frequently observed during disease flares and correlate with heightened inflammatory activity, with specific clades enriched exclusively in IBD patients [35]. Alterations in mucosa-associated microbial communities are also clinically relevant; for example, increased *Haemophilus parainfluenzae* abundance has been linked to ulcerative colitis severity and remission patterns, with direct detection in intestinal biopsies [36]. Conversely, some pediatric cohorts at disease onset have shown elevated *F. prausnitzii*, suggesting a more context dependent role for this species [36].

Dysbiosis also involves depletion of beneficial mucosal species such as *Bifidobacterium longum*, *B. bifidum* and *B. adolescentis*, which play essential roles in epithelial renewal, barrier integrity and SCFA production [7,28]. Distinct microbial signatures further differentiate Crohn’s disease from ulcerative colitis. Crohn’s disease is characterized by increased *Actinomyces*, *Veillonella* and *Escherichia coli*, whereas ulcerative colitis typically shows enrichment of *Eubacterium rectale* and decreased *Akkermansia muciniphila* [37]. Chronic inflammation, oxidative stress and impaired epithelial barrier function create an ecological niche favoring facultative anaerobes such as Enterobacteriaceae [9]. Alterations in tight junction regulation further promote microbial translocation and sustained inflammatory signaling [11].

Conventional therapies, including mesalamine, may also influence microbial composition. Mesalamine has been associated with reductions in *Escherichia*/Shigella and modest increases in *Enterococcus*, reflecting treatment-specific microbial effects [38].

Diet and microbiota directed therapies have gained increasing attention as adjuncts in IBD management. While current treatments primarily target inflammation and barrier dysfunction, their limitations underscore the need for complementary microbiota-focused strategies [39]. Dietary interventions can modulate microbial diversity and SCFA availability, though long term clinical implications require more investigation [40]. Prebiotic supplementation may enhance epithelial barrier function, decrease pro-inflammatory cytokines and inhibit pathogenic bacterial proliferation [41]. Exclusive enteral nutrition, the only evidence based dietary therapy for pediatric Crohn’s disease, effectively induces remission but is hindered by limited adherence and rapid symptom recurrence after reintroducing regular food [42]. Fecal microbiota transplantation has shown promise in pediatric IBD, although lack of standardized protocols currently limits its broader use [43]. Nutrient deficiencies remain common due to malabsorption and dietary restrictions, necessitating ongoing monitoring and individualized nutritional support [44].

Host genetic susceptibility also interacts with the microbiota to shape disease phenotype. Genetic polymorphisms can influence microbial community structure, while dysbiosis may activate pathological immune responses in genetically predisposed individuals [17]. These interactions highlight the potential of microbial biomarkers for predicting disease activity and therapeutic response [26]. Despite considerable research, it remains unclear whether microbial alterations are sustained or specifically treatment driven, emphasizing the need for longitudinal evaluations of therapeutic effects on the microbiota [45].

An impaired ability of the host immune system to differentiate pathogenic from commensal bacteria has been described as a contributing mechanism in IBD and may perpetuate inflammation and maintain dysbiosis [45]. Chronic inflammation, oxidative stress, epithelial injury, dietary patterns and genetic predisposition collectively shape a distorted microbial ecosystem and contribute to heterogeneous clinical outcomes.

Pediatric IBD represents a unique clinical context in which microbiota–host interactions occur during critical periods of immune and microbial maturation. Early-life dysbiosis may therefore have long-term implications for disease course, remission stability, and transition into adulthood. In this setting, identifying reproducible microbial patterns and quantifying dysbiosis severity may be particularly relevant for guiding personalized therapeutic strategies.

This study has several limitations. Microbiota profiling was based on fecal samples, which may not fully represent mucosa-associated microbial communities. The uniform administration of sodium butyrate restricts evaluation of its independent effects. Subgroup analyses may have been underpowered. Additionally, the AI-assisted platform provides semi-quantitative rather than full metagenomic data, limiting the interpretation of microbial function.

Future research should integrate microbial, metabolic, immune and genetic markers to advance predictive modeling of treatment response. Longitudinal studies examining microbiota trajectories under biologic therapy, dietary interventions, prebiotics and SCFA targeted strategies may support the development of personalized therapeutic approaches for pediatric IBD.

Overall, the findings of this study reinforce the central role of intestinal dysbiosis in pediatric inflammatory bowel disease and highlight the value of microbiota-derived indices as complementary tools for assessing disease activity and therapeutic response.

## 5. Conclusions

Pediatric IBD is associated with marked intestinal dysbiosis, most pronounced in Crohn’s disease. Biologic therapy and sodium butyrate supplementation contributed to partial restoration of microbial balance, while strong correlations between microbial indices and dysbiosis scores reinforce their value as complementary markers of gut health. These findings highlight the central role of microbial imbalance in disease mechanisms and support the potential of microbiota targeted interventions to improve clinical outcomes. Further studies are required to validate these results and clarify their therapeutic implications.

## Figures and Tables

**Table 1:** Demographic and clinical characteristics of the study population.

Characteristic	Category	Value (*n* = 60)
Age (years)	Mean	13.77
Sex	Male	38 (63.3%)
	Female	22 (36.7%)
Diagnosis	Crohn’s disease (CD)	36 (60%)
	Ulcerative colitis (UC)	24 (40%)
Delivery mode	Cesarean section	36 (60%)
	Vaginal delivery	24 (40%)
Early-life feeding	Exclusive breastfeeding	15 (25%)
	Exclusive formula	15 (25%)
	Mixed feeding (breast milk + formula)	28 (46.7%)
	Breast milk + cow’s milk	2 (3.3%)
Initial treatment	Biologic therapy	17 (28.3%)
	Azathioprine	17 (28.3%)
	Biologic + azathioprine	16 (26.7%)
	5-ASA + corticosteroids	3 (5%)
	5-ASA alone	3 (5%)
	Corticosteroids alone	3 (5%)
	Triple therapy (biologic + azathioprine + 5-ASA)	1 (1.7%)

**Table 2:** Mean dysbiosis scores across clinical and therapeutic subgroups.

Comparison	Group	*n*	Mean Dysbiosis Score (±SD)	Interpretation
Disease type	Crohn’s disease	36	9.65	Higher dysbiosis severity
	Ulcerative colitis	24	8.42	Milder microbiota alteration
Therapy (overall)	With biologic therapy	28	8.8	Suggests microbiota protection
	Without biologic therapy	32	9.5	More severe dysbiosis
Crohn’s subgroup	Biologic	18	9.56	Lower dysbiosis score
	Non-biologic	18	9.78	Higher dysbiosis score
UC subgroup	Biologic	10	9.0	Slightly higher, non-significant
	Non-biologic	14	8.0	Slightly lower
Corticosteroid therapy	Yes	3	9.3	Minimal difference
	No	57	9.0	—
Feeding type	Exclusive breastfeeding	15	8.6	Protective effect
	Formula feeding	15	9.4	Higher dysbiosis
	Mixed feeding	28	9.1	Intermediate values

**Table 3:** Mean number of pathobionts per patient by diagnosis and treatment.

Comparison	Group	*n*	Pathobionts/Patient (Mean ± SD)
Disease type	Crohn’s disease	36	2.76 ± 1.44
	Ulcerative colitis	24	2.50 ± 1.88
Treatment (all patients)	With biologic therapy	28	2.43 ± 1.70
	Without biologic therapy	30	2.87 ± 1.55
Crohn’s subgroup	Biologic	18	2.56
	Non-biologic	18	3.00
UC subgroup	Biologic	10	2.20
	Non-biologic	14	2.71

**Table 4:** Chi-square analysis results between diagnosis, treatment, and bacterial profile.

Analysis	Main Finding	*p*-Value	Interpretation
*Bilophila wadsworthia* (CD vs UC)	More frequent in Crohn’s disease	0.024	Significant association
*Clostridioides difficile* (CD vs UC)	More frequent in Crohn’s disease	0.032	Significant association
*Ruminococcus torques* (CD vs UC)	More frequent in ulcerative colitis	0.044	Significant association
≥3 pathobionts (CD vs UC)	More frequent in Crohn’s disease	0.028	Significant association
≥3 pathobionts (biologic vs non-biologic)	Slightly lower in biologic group	0.35	Not statistically significant

**Table 5:** Mean microbial indices by diagnosis (N = 60).

Parameter	Crohn’s Disease (*n* = 36)—Mean ± SD	Ulcerative Colitis (*n* = 24)—Mean ± SD	*p*-Value
Organism of Interest	7.87 ± 1.36	7.42 ± 1.52	0.28
GMI	20.87 ± 3.26	21.71 ± 3.29	0.46

Interpretation: Microbial indices were similar between Crohn’s disease and ulcerative colitis, with slightly higher values in Crohn’s disease, suggesting a trend toward more pronounced dysbiosis.

**Table 6:** Mean microbial indices by biologic treatment (N = 60).

Parameter	With Biologic Therapy (*n* = 32)—Mean ± SD	Without Biologic Therapy (*n* = 28)—Mean ± SD	*p*-Value
Organism of Interest	7.12 ± 1.42	8.02 ± 1.33	0.048 *
GMI	20.12 ± 3.22	22.17 ± 3.38	0.039 *

* *p* < 0.05 was considered statistically significant. Interpretation: Patients receiving biologic therapy displayed significantly lower microbial index values, suggesting a beneficial effect of biologic agents on intestinal microbial balance.

**Table 7:** Spearman correlation between microbial indices (N = 60).

Correlation	r	*p*-Value	Interpretation
Organism of Interest ↔ GMI	+0.68	0.001 **	Strong positive, statistically significant correlation

** *p* < 0.01 indicates statistical significance. Interpretation: The strong positive correlation confirms that both indices reflect similar aspects of microbial dysregulation and mutually reinforce their diagnostic reliability.

**Table 8:** Dysbiosis score by diagnosis (N = 60).

Diagnosis	*n* (%)	Mean ± SD	*p*-Value
Crohn’s disease	36 (60%)	9.65 ± 1.44	0.037 *
Ulcerative colitis	24 (40%)	8.42 ± 1.88	—

* *p* < 0.05 was considered statistically significant. Interpretation: Patients with Crohn’s disease presented significantly higher dysbiosis scores.

**Table 9:** Dysbiosis score by treatment type.

Treatment	*n* (%)	Mean ± SD	*p*-Value
Biologic therapy	28 (47%)	8.8 ± 1.70	0.17
Non-biologic therapy	32 (53%)	9.5 ± 1.55	—

Interpretation: Although not statistically significant, biologic therapy showed a tendency toward lower dysbiosis.

**Table 10:** Distribution of dysbiosis severity.

Severity	*n*	%
Mild	10	16.7%
Moderate	22	36.6%
Severe	28	46.7%

**Table 11:** Microbial indices (Organism of Interest and GMI).

Diagnosis	Organism of Interest (Mean ± SD)	GMI (Mean ± SD)	*p*-Value
Crohn’s disease	7.87 ± 1.36	20.87 ± 3.26	0.42
Ulcerative colitis	7.42 ± 1.52	21.71 ± 3.29	—

**Table 12:** Correlations between dysbiosis score and microbial indices.

Correlation	*r*	*p*-Value	Interpretation
Dysbiosis ↔ Organism of Interest	+0.68	<0.01 *	Strong positive correlation
Dysbiosis ↔ GMI	+0.59	<0.01 *	Significant positive correlation
Organism of Interest ↔ GMI	+0.72	<0.01 *	High concordance

* *p* < 0.05 was considered statistically significant.

**Table 13:** Evolution of microbial indices after butyrate (N = 60).

Parameter	Before (Mean ± SD)	After (Mean ± SD)	Δ Mean ± SD	*p*-Value
Organism of Interest	8.65 ± 1.36	7.97 ± 1.31	−0.68 ± 1.02	0.021 *
GMI	23.65 ± 3.26	21.61 ± 3.24	−2.04 ± 2.85	0.017 *

* *p* < 0.05 was considered statistically significant.

**Table 14:** Differences by diagnosis.

Diagnosis	Organism of Interest	GMI	*p*-Value
Crohn’s disease	−0.79 ± 1.08	−2.31 ± 2.77	0.031 *
Ulcerative colitis	−0.52 ± 0.95	−1.66 ± 2.93	0.083

* *p* < 0.05 was considered statistically significant.

**Table 15:** Differences by biologic treatment.

Treatment	Organism of Interest	GMI	*p*-Value
Biologic	−0.83 ± 0.97	−2.25 ± 2.65	0.029 *
Non-biologic	−0.51 ± 1.06	−1.77 ± 3.01	

* *p* < 0.05 was considered statistically significant.

## Data Availability

The data supporting the findings of this study are available from the corresponding author upon reasonable request. The data are not publicly available due to ethical restrictions and the protection of patient confidentiality.

## Supplementary Materials

The following supporting information can be downloaded at https://www.mdpi.com/article/10.3390/jcm15041589/s1, Supplementary Material S1. Workflow of AI-assisted microbiota profiling.
