# Supplementary material for: Gut Microbiota Alterations and Dysbiosis Patterns in Pediatric Inflammatory Bowel Disease: Clinical Correlations and Therapeutic Impact
Source: J Clin Med. 2026 Feb 18;15(4):1589. doi: 10.3390/jcm15041589 (PMC12941501; doi:10.3390/jcm15041589)
Supplement: Supplementary file 1 [file jcm-15-01589-s001.zip › jcm-4119567-supplementary.pdf]

Supplementary Material S1
Workflow of AI-assisted microbiota profiling

Fecal samples were collected under standard clinical conditions. DNA extraction and 16S rRNA sequencing were performed in a certified reference laboratory. Sequencing data underwent bioinformatic preprocessing and taxonomic assignment. An AI-assisted platform (NostraBiome, Arad, Romania) was subsequently used for pattern recognition and classification of dysbiosis profiles according to the analytical workflow of the reference laboratory. Based on these analyses, semi-quantitative microbiota-derived indices, including the Gut Microbiota Index and the Organism of Interest metric, were generated for clinical interpretation.
